# Coinfection with Different *Trypanosoma cruzi* Strains Interferes with the Host Immune Response to Infection

**DOI:** 10.1371/journal.pntd.0000846

**Published:** 2010-10-12

**Authors:** Claudiney Melquíades Rodrigues, Helder Magno Silva Valadares, Amanda Fortes Francisco, Jerusa Marilda Arantes, Camila França Campos, Andréa Teixeira-Carvalho, Olindo Assis Martins-Filho, Márcio Sobreira Silva Araujo, Rosa Maria Esteves Arantes, Egler Chiari, Glória Regina Franco, Carlos Renato Machado, Sérgio Danilo Junho Pena, Ana Maria Caetano Faria, Andréa Mara Macedo

**Affiliations:** 1 Departamento de Bioquímica e Imunologia, Instituto de Ciências Biológicas, Universidade Federal de Minas Gerais, Belo Horizonte, Brazil; 2 Laboratório de Imunopatologia, Núcleo de Pesquisas em Ciências Biológicas, Instituto de Ciências Exatas e Biológicas, Universidade Federal de Ouro Preto, Ouro Preto, Brazil; 3 Laboratório de Biomarcadores de Diagnóstico e Monitoração, Centro de Pesquisas René Rachou, Fundação Oswaldo Cruz, Belo Horizonte, Brazil; 4 Departamento de Patologia, Instituto de Ciências Biológicas, Universidade Federal de Minas Gerais, Belo Horizonte, Brazil; 5 Departamento de Parasitologia, Instituto de Ciências Biológicas, Universidade Federal de Minas Gerais, Belo Horizonte, Brazil; René Rachou Research Center, Brazil

## Abstract

A century after the discovery of *Trypanosoma cruzi* in a child living in Lassance, Minas Gerais, Brazil in 1909, many uncertainties remain with respect to factors determining the pathogenesis of Chagas disease (CD). Herein, we simultaneously investigate the contribution of both host and parasite factors during acute phase of infection in BALB/c mice infected with the JG and/or CL Brener *T. cruzi* strains. JG single infected mice presented reduced parasitemia and heart parasitism, no mortality, levels of pro-inflammatory mediators (TNF-α, CCL2, IL-6 and IFN-γ) similar to those found among naïve animals and no clinical manifestations of disease. On the other hand, CL Brener single infected mice presented higher parasitemia and heart parasitism, as well as an increased systemic release of pro-inflammatory mediators and higher mortality probably due to a toxic shock-like systemic inflammatory response. Interestingly, coinfection with JG and CL Brener strains resulted in intermediate parasitemia, heart parasitism and mortality. This was accompanied by an increase in the systemic release of IL-10 with a parallel increase in the number of MAC-3^+^ and CD4^+^ T spleen cells expressing IL-10. Therefore, the endogenous production of IL-10 elicited by coinfection seems to be crucial to counterregulate the potentially lethal effects triggered by systemic release of pro-inflammatory mediators induced by CL Brener single infection. In conclusion, our results suggest that the composition of the infecting parasite population plays a role in the host response to *T. cruzi* in determining the severity of the disease in experimentally infected BALB/c mice. The combination of JG and CL Brener was able to trigger both protective inflammatory immunity and regulatory immune mechanisms that attenuate damage caused by inflammation and disease severity in BALB/c mice.

## Author Summary

Chagas disease, a life-long parasitic disease caused by the flagellate protozoan *Trypanosoma cruzi*, was discovered a century ago by the Brazilian physician Carlos Chagas, and remains one of the most neglected tropical diseases, affecting 13 million people in Latin America. Disease is characterized by distinct clinical courses, varying from asymptomatic to severe forms with damage to heart and/or gastrointestinal tract. The causes of the different clinical manifestations are not completely understood, but they certainly involve both parasite and host features. In this study, the authors analyzed immune response of BALB/c mice to infection with two different *T. cruzi* populations. One of them (JG) caused low parasitism and low levels of pro-inflammatory mediators associated with no clinical manifestation of the disease. The other (CL Brener) caused severe disease, high mortality and high levels of pro-inflammatory mediators. The coinfection, however, triggered singular regulatory immune mechanism(s) that attenuated damage caused by inflammation and disease severity that are typical of the single infection with CL Brener. As mixed infection is naturally found in patients in endemic areas, these results can explain, at least in part, the complexity of the immune responses and consequently the various clinical manifestations of the disease.

## Introduction

Chagas disease (CD), a life-long complex illness caused by the protozoan parasite *Trypanosoma cruzi*, was firstly described by Carlos Chagas in 1909, but it is still acknowledged by the World Health Organization (WHO) as one of the most important neglected tropical diseases and as a significant public health problem in Central and South America [1]. *T. cruzi* is transmitted to humans and other susceptible hosts mainly through contact with the feces of infected blood-feeding triatomines, but alternative routes such as blood transfusion, organ transplant, vertical transmission (congenital) or ingestion of contaminated food (oral transmission) are presently more important in the current context of CD.

Despite one century of research, the most intriguing challenge to understanding the physiopathology of CD still lies in the complex host-parasite interrelationship. From the clinical point of view, *T. cruzi* infections progress in two phases. Patent parasitemia and parasitism in a wide variety of host cells characterize the acute phase of disease. This phase normally passes unnoticed because the signs and symptoms are similar to those of most common infections: fever, swollen lymph nodes, hepato- and/or splenomegaly. Sterile immunity is rarely achieved after *T. cruzi* infection, and most of the patients that survive the acute phase remain in a life-long asymptomatic state (indeterminate form) during the chronic phase of infection. However, a significant percentage of these patients (about 40%) develop deadly clinical forms of the disease up to 20 years after the first contact with the parasite, as a result of progressive tissue damage mainly involving the esophagus, colon and/or heart. On average, 5–10% of the *T. cruzi* infected individuals develop the digestive form of the disease and 30–40% develop cardiomyopathy (cardiac form), the most severe clinical manifestation of CD. The associated cardio-digestive form is observed in 2–3% of the patients [2].

The severity and prevalence of the different clinical forms of CD vary among different regions [2], but the cause of this clinical and epidemiological heterogeneity is a puzzling and yet unresolved question. Despite many uncertainties, it is more and more clear that the pathogenesis of CD is very complex and is a multifactorial trait influenced by several factors related to the parasite, the host and maybe also the environment [3]–[10].

Concerning to parasite related factors, there is extensive and well-characterized intraspecific genetic diversity in *T. cruzi*, which has been demonstrated by different biological, biochemical and molecular approaches [11]. The coexistence of mixed infections in vertebrate and invertebrate hosts has also been demonstrated in natural situations [12]–[14] and this certainly plays an important role in the context of the pathogenesis of CD. For instance, distinct parasite populations have been found in different tissues (blood, esophagus and heart) of the same chronically infected patients [14], [15], suggesting that specific tissue tropism of the parasite is one of the major factors determining the pathology of this illness. Similarly, *T. cruzi* genetic variability was shown to be an important factor influencing tissue tropism and pathogenesis in BALB/c mice double-infected with an artificial mixture of JG (*T. cruzi* II) and Col1.7G2 (*T. cruzi* I) monoclonal populations [3]. A clear difference in tissue tropism was observed after three months post-infection: the Co1.7G2 clone predominated over the JG strain in the rectum, diaphragm, esophagus, and blood, while a striking amount of the JG strain was observed in the heart muscles of coinfected mice. Intriguing results were also observed by Franco *et al.* (2003) in studying the effects of coinfection with two *T. cruzi* populations exhibiting opposing virulence and pathogenicity in Holtzman rats: the CL Brener (*T. cruzi* VI) clone, which induces severe and diffuse myocarditis with high mortality, and the JG strain, which causes moderate acute myocarditis with no mortality. Although less virulent when compared to CL Brener in single infections, the JG strain was the only parasite detected in the rat tissues at the end of the acute phase of the double infection, in contrast to the results observed in the single infection protocols [8].

Concerning to host related factors, it is also well accepted that genetic polymorphisms associated with the host's immune response have an essential role in determining the course of *T. cruzi* infection. In fact, there is evidence that changes in cytokine expression patterns during the course of infection play an important role in the disease outcome [7]. For instance, *in vitro* exposure to *T. cruzi* trypomastigotes induces higher expression of IL-10 in monocytes isolated from indeterminate patients relative to cardiac patients, suggesting an immunological imbalance among patients with the cardiac clinical form of CD [16]. The lower expression of IL-10 among cardiac patients was associated with occurrence of a polymorphism in the promoter region of the IL-10 gene [5]. Furthermore, associations of polymorphisms in the genes for BAT-1 and NFκB with the development of cardiomiopathy were also described for CD in the Brazilian population [10], [17]. Host genetic factors are also involved in determining parasite tissue tropism in experimental CD. Andrade *et al.* (2002) clearly demonstrated that the genetic background of mouse strains (BALB/c, DBA-2, C57BL/6, and Swiss) influences the differential tissue distribution of JG and Col1.7G2 populations in double-infected animals [4]. Subsequently, using congenic mice, Freitas *et al.* (2009) identified MHC-associated genes as those mainly involved in determining the differential tissue tropism of these two parasite populations [9].

In conclusion, there are many studies alternatively demonstrating the importance of parasite or host immune response factors influencing the pathogenesis of the CD, but the present work is probably the first one that simultaneously investigates the mechanism and the contribution of both parts. Herein, we assess the parasitemia, body weight evolution, survival rate, different hematological parameters, heart parasitism and histopathology, and heart differential tissue tropism. We also perform quantitative analyses of serum cytokines and nitric oxide as well as flow cytometry analyses of spleen cells during the acute phase of infection with the JG and/or CL Brener in BALB/c mice. We clearly demonstrate that coinfection with JG and CL Brener is able to trigger both protective inflammatory immunity and regulatory immune mechanisms that are capable of both attenuate damage caused by inflammation and disease severity induced by single infection with CL Brener in BALB/c mice.

## Materials and Methods

All animals were handled in strict accordance with good animal practice as defined by the Internal Ethics Commitee in Animal Experimentation of the Universidade Federal de Minas Gerais (UFMG), Belo Horizonte (BH), Minas Gerais (MG), Brazil (CETEA/UFMG - Protocol no. 5/2007).

### Animals and infection

Six to eight-week-old inbred male BALB/c mice, bred and maintained in the animal breeding units at the Instituto de Ciências Biológicas (ICB/UFMG), or Centro de Pesquisas René Rachou/Fundação Oswaldo Cruz (CPqRR/FIOCRUZ), both in BH/MG, Brazil, were used. We used two different *T. cruzi* populations: the JG strain (*T. cruzi* II), which was isolated from a chagasic patient with mega esophagus, and the CL Brener clone (*T. cruzi* VI), which was obtained from CL strain isolated from a *Triatoma infestans* specimen. Parasite major lineages were identified as recently recommended by an expert committee [18]. Both *T. cruzi* populations were maintained by intraperitoneal (i.p.) inoculation of infective blood trypomastigotes in Swiss mice. Prior genetic characterization of *T. cruzi* populations used in this work (Table 1) was done by typing seven polymorphic microsatellite loci [19], [20], and the genes for 24Sα rDNA [21] and cytochrome oxidase subunit II (COII) [22]. For BALB/c mice infections, infective blood trypomastigotes were obtained from retroorbital plexus of JG or CL Brener infected Swiss mice. Both trypomastigote populations were counted and diluted in LIT medium. BALB/c mice were i.p. inoculated with 0.10 ml of a suspension containing 100 trypomastigotes of JG or CL Brener (single infection), or a mixture of 50 trypomastigotes of each (double infection). Age- and sex-matched non-infected BALB/c mice were used as controls. Experimental groups consisted of three, six or twelve mice. The experiments were repeated at least twice.

**Table 1:** Genetic characterization of nuclear and mitochondrial markers of the JG and CL Brener *T. cruzi* strains.

*Marker*	*JG strain*	*CL Brener clone*
*Major lineage* a	*T. cruzi II*	*T. cruzi VI*
*COII* b	C	B
*rDNA 24Sα* c	1	1
*MCLE01*	134/136	130/130
*MCLF10*	182/182	182/194
*SCLE10*	273/273	237/275
*SCLE11*	145/149	153/153
*TcAAAT6*	271/275	263/263
*TcTAC15*	99/99	129/141
*TcTAT20*	190/217	181/223

a Major lineages nomenclature for *T. cruzi* strains in accordance with Zigales *et al.* (2009).

b *Alu*I restriction fragment length polymorphism (RFLP) of the *T. cruzi* cytochrome oxidase subunit II (COII) gene (Freitas *et al.*, 2006).

c *rDNA 24Sα* group of the *T. cruzi* as defined by Souto *et al.* (1996).

### Parasitemia, body weight and survival assessment

Parasitemia was assessed by counting the bloodstream form of parasites in 5.0 µl of tail vein blood of JG and/or CL Brener infected mice, on alternate days from the 5th day p.i. until the time point that the parasites became undetectable [23]. Data were expressed as number of trypomastigotes per milliliter of blood. Survival was determined by daily inspection post-infection (p.i.), and mice were weighed on alternate days to monitor the systemic repercussions during the course of infection.

### Hematological analyses

At 7, 14 and 21 days post-infection, mice were bled from the axillary plexus under xylazine/ketamine anesthesia and peripheral blood (PB) was collected with anticoagulant for hematological analyses or without anticoagulant for serum cytokine and nitric oxide (NO) assays. The hematological parameters (leukocytes, red blood cells, platelets, hematocrit and hemoglobin) were determined using the ABX Micros ABC Vet automatic system (Horiba ABX diagnostics, Montpellier, France). Differential leukocyte counts were determined under the oil immersion objective (100×), using standard morphological criteria, in peripheral blood smears stained with May-Grünwald-Giemsa, and the absolute number of each leukocyte subtype per ml of PB was determined.

### Heart histopathology, morphometric analysis and parasitism

To compare the effects of JG and/or CL Brener infection on influx of inflammatory cells in the heart, we analyzed the intensity of myocarditis morphometrically. For this experiment, animals were euthanized by cervical displacement, and the hearts were removed and sliced transversally at 7, 14 and 21 days p.i. The apical half of each heart was washed in phosphate-buffered saline (PBS) and stored in absolute ethanol at 4°C for PCR assays; the heart bases were fixed in 4% phosphate-buffered formaldehyde and used for histopathology. After 24 hours of fixation, the tissues were paraffin-embedded, and three 5-µm thick, semi-consecutive sections were obtained and stained by hematoxylin-eosin (H&E). Heart inflammation was assessed in the left ventricle free wall. For quantitative analyses, ten fields from each of the three semi-consecutive sections were randomly captured with the 40× objective, corresponding to a total myocardium area of 234.376 µm^2^. Images were captured at a resolution of 1392×1040 pixels with a Cool SNAP-Pro cf Collor microcamera (Media Cybernetics, Bethesda, MD, EUA) and transferred to a computer using Image-Pro Express version 4.0 software for Windows (Media Cybernetics). After proper calibration, captured images were analyzed with KS300 software (Zeiss, Jena, Germany). The nucleus area from each cell presented in the analyzed fields was digitalized and automatically measured in µm^2^. The results were expressed by nucleus area/total area ratio. Heart parasitism was evaluated by counting the number of parasite nests in three semi-consecutive sections as visualized by light microscopy with a 40× objective. PCR was additionally performed in parallel samples.

### Detection and characterization of parasites in heart tissue samples by PCR

Detection of parasites in heart tissue samples was performed by specific PCR amplification of a fragment of about 330 bp from variable regions of minicircle kinetoplast DNA (kDNA) molecules of *T. cruzi*, as previously described [3] with some modifications. Tissue samples, stored in absolute ethanol, were fragmented and submitted to alkaline lysis, as follows: the fragmented samples were boiled in the presence of 50 mM NaOH for 10 min, and after neutralization with 130 mM Tris-HCl pH 7, samples were 10-fold diluted in sterile Milli-Q water and used as the DNA template for PCR. Samples from uninfected BALB/c mice were used as a negative control. PCR was carried out in a final volume of 20 µl containing 1.5 mM MgCl_2_, Green Go*Taq* Reaction Buffer pH 8.5 (Promega, Madison, Wisconsin, USA), dNTPs at 250 µM, primers (S35: 5′-AAATAATGTACGGGKGAGATGCATGA-3′ and S36: 5′-GGTTCGATTGGGGTTGGTGTAATATA-3′) at 1.0 µM, 1.0 U of Go*Taq* DNA Polymerase (Promega) and 1.0 µl of 10-fold diluted alkaline lysis products. Amplification was performed in a PT100 thermocycler (MJ Research) using an initial denaturation step at 94°C for 5 min followed by 35 amplification cycles including an annealing step at 60°C, extension at 72°C and denaturation at 94°C, each for 1 min. At the end, the extension step was extended to 10 min. The PCR products were visualized on a 6% polyacrylamide gel using silver staining, as previously described (Santos et al., 1993). Differential tissue tropism of both *T. cruzi* populations was assessed by analyzing the LSSP-PCR profiles and one of the previously typed polymorphic microsatellite loci (*TcAAAT6*) in positive tissue samples from double-infected mice.

### Assessment of differential tissue tropism in the heart by LSSP-PCR

**Figure 1:**
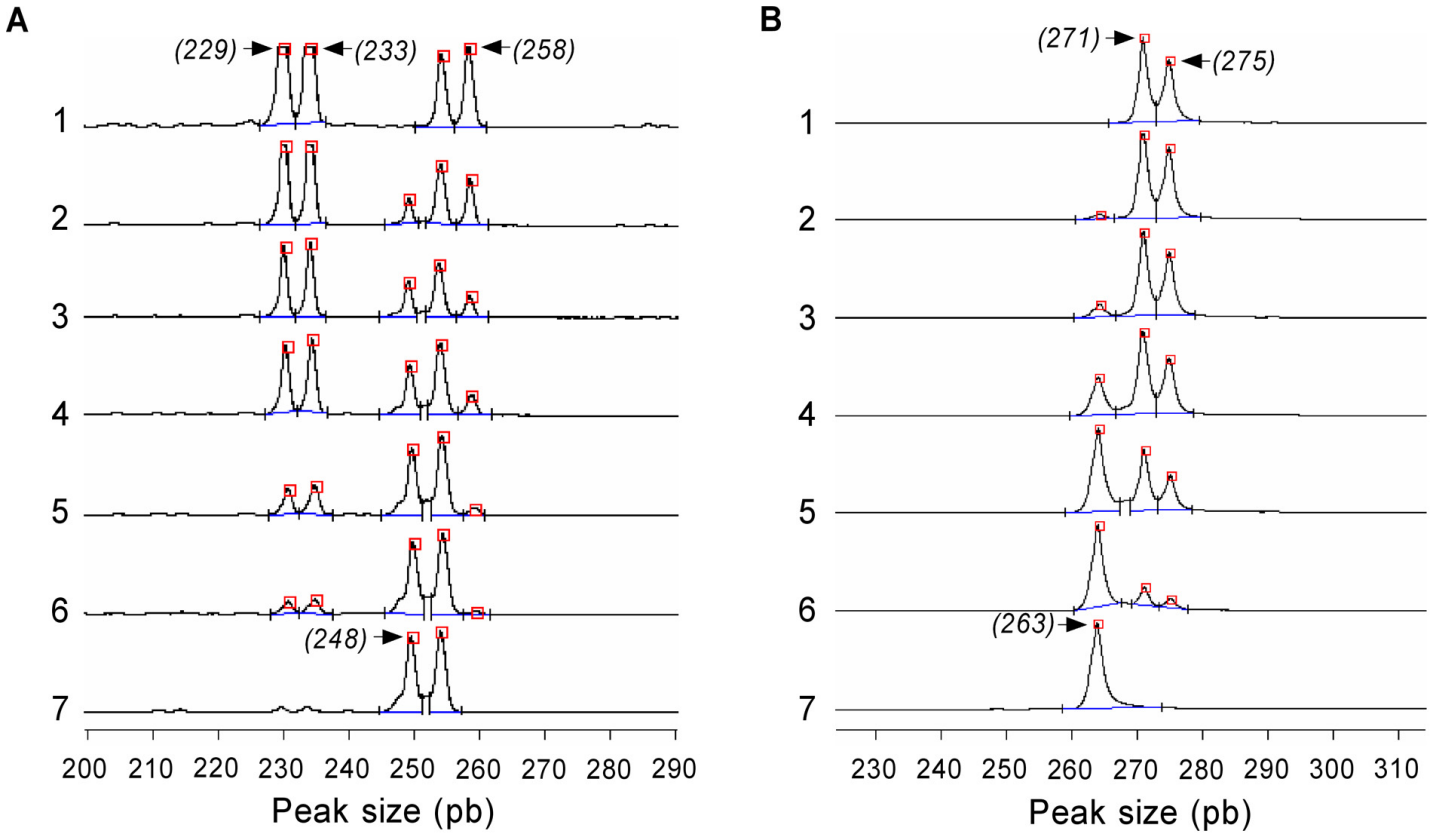
Electrofluorograms obtained by LSSP-PCR and polymorphic microsatellite (locus *TcAAAT6*) analyses of artificial mixtures of DNA from JG and CL Brener. Genomic DNA samples from JG (lane 1) and CL Brener (lane 7) were mixed at different proportions (JG/CL Brener: 9/1 (lane 2), 3/1 (lane 3), 1/1 (lane 4), 1/3 (lane 5) and 1/9 (lane 6)), as illustrated in the figures, and subjected to PCR assays. Fluorescent products of LSSP-PCR (A) and *TcAAAT6* (B) were loaded in each lane in a 6% polyacrylamide gel under denaturing conditions in an automatic laser fluorescent (ALF) DNA sequencer. The numbers at the peaks refer to the size of amplicons and arrows indicate specific peaks from JG or CL Brener, which were used to produce the standard curves.

The relative proportions of JG and CL Brener in the positive heart tissue samples obtained from double-infected mice were estimated using the LSSP-PCR assay, as previously described [3] with some modifications. For this, kDNA amplicons were subjected to electrophoresis on an ethidium bromide stained 1.5% agarose gel (1.0% agarose, 0.5% low melting point agarose) at 100 V for 1 h 30 min. The DNA bands corresponding to the 330-bp amplicons from variable regions of *T. cruzi* kDNA minicircles were purified from the gel, diluted 10-fold in sterile Milli-Q water, and subjected to a second PCR assay at low stringency, using a single fluorescent primer. The PCR was carried out in a final volume of 10 µl containing 1.5 mM MgCl_2_, Colorless Go*Taq* Reaction Buffer pH 8.5 (Promega), dNTPs at 50 µM, fluorescent primer (S35G*: 5′-*Fluorescein* ATGTACGGGGGAGATGCATGA-3′) at 4.5 µM, 1.6 U of Go*Taq* DNA Polymerase (Promega) and 1.0 µl of a solution containing the ∼330-bp DNA fragments prepared as described above. Amplification was performed in a PT100 thermocycler (MJ Research) as follows: an initial denaturation step at 94°C for 5 min, followed by 40 amplification cycles with the annealing step at 30°C, extension at 72°C, and denaturation at 94°C, each for 1 min. The final extension step was extended to 10 min. To determine the DNA fragment sizes, the LSSP-PCR products were analyzed by 6% polyacrylamide gel electrophoresis under denaturing conditions (8 M urea) in an Automatic Laser Fluorescent (ALF) sequencer (GE Healthcare, Milwaukee, Wisconsin, USA) followed by data analysis using the Allelelocator software (GE Healthcare). Areas under specific peaks from JG and CL Brener curves were used to estimate the relative proportions of each population in reference to a standard curve, as previously described [3]. Briefly, genomic DNA samples from JG and CL Brener were mixed in different proportions (JG/CL Brener: 9/1 (lane 2), 3/1 (lane 3), 1/1 (lane 4), 1/3 (lane 5) and 1/9 (lane 6)), and subjected to PCR assays. Fluorescent products of PCR were loaded into a 6% polyacrylamide gel under denaturing conditions in an automated DNA sequencer. The proportions of the sum of areas under specific peaks (Figure 1A) of each population were used to construct a standard curve. The standard curve was obtained using GraphPad Prism 5.00 software (GraphPad Software, San Diego, California, USA) by point-to-point analysis without the choice of any specific model. We used 96 points calculated with the x values (relative proportion of JG/CL Brener) ranging from 0.0 to 0.9 for building the standard curve (data not shown).

### Assessment of differential tissue tropism in the heart by *TcAAAT6* microsatellite locus genotyping

The relative proportions of JG and CL Brener in the hearts of the double infected mice were also assayed by genotyping the *TcAAAT6* microsatellite locus. To achieve that, a full nested PCR protocol was used, as previously described [20] with some modifications. Briefly, PCR was performed in a final volume of 15 µl containing 10 mM Tris-HCl pH 9.0, 50 mM KCl, 0.1% Triton X-100 (Buffer B, Promega), 2.5 mM MgCl_2_ (Promega), 0.5 U of *Taq* DNA Polymerase (Promega), dNTPs at 250 µM, primers (*TcAAAT6*ex-forward 5′-ACGCACTCTCTTTGTTAACAG-3′ and *TcAAAT6*ex-reverse 5′-CCGACAACGATGACAGCAAT-3′) at 0.3 µM and 1.0 µl of DNA template (10-fold diluted alkaline lysis products). Amplification was performed in a PT100 thermocycler (MJ Research) using the step-down protocol modified for amplification of *T. cruzi* DNA as follows: an initial denaturation step at 94°C for 5 min; annealing at 58°C for 30 s; extension at 72°C for 1 min and a denaturation step at 94°C for 30 s. After every five cycles, the annealing temperature was decreased by two degrees to 55, 53, 51 and finally 48°C. At this last temperature, the number of cycles was increased to 15, followed by a final extension step at 72°C for 10 min. A second round of amplification was performed in same conditions described above but with inner primers (*TcAAAT6*-forward 5′-*Fluorescein*GCCGTGTCCTAAAGAGCAAG-3′ and *TcAAAT6*-reverse 5′-GGTTTTAGGGCCTTTAGGTG-3′). For the second PCR round, 10% of the amplified products obtained in the first PCR round were used as the DNA template. The determination of allele sizes was performed as described above. Areas under specific peaks from JG and CL Brener were used to estimate the relative proportions of each population by reference to a standard curve (Figure 1B), as described above.

### Quantitative analysis of serum cytokines

For cytokine analysis, serum samples were collected as previously described and stored at −20°C until used. Cytokines (IL-2, IL-4, IL-5, IL-6, IL-10, IL-12p70, IFN-γ, CCL2 and TNF-α) were measured with BD CBA Mouse Cytokine assay kits according to the manufacturer's specifications (BD Biosciences, CA, USA).

### Serum nitrite evaluation

Serum nitric oxide (NO), an oxidation product of arginine by NO synthase, was measured as nitrite (NO_2_^−^), the stable product of reactive nitrogen intermediates, at 7, 14 and 21 days p.i. in samples collected as described above. Serum nitrite levels were assessed using the Griess reaction, after deproteination of samples with 1 M ZnCl_2_. Nitrite concentrations were determined by extrapolation from a standard curve constructed using various concentrations of sodium nitrite (NaNO_2_^−^), and the results were expressed in µM.

### Immunofluorescence staining and flow cytometry analysis of spleen cells

Spleen samples were collected at 7, 14 and 21 days p.i. in RPMI-1640 (GIBCO, Grand Island, NY, USA). Spleen cell suspensions were prepared as previously described [24] and kept on ice. Cells were counted and incubated for 12 h at 37°C in a 5% CO_2_ humidified incubator and re-incubated again for more 4 h in the presence of 10 µg/ml brefeldin A (BFA) (Sigma, St. Louis, MO, USA), in the same conditions. Cell samples were then treated with 2.0 mM ethylenediaminetetraacetic acid (EDTA) (Sigma, St. Louis, MO, USA) for 10 min at room temperature and washed once with FACS buffer (PBS with 0.5% of bovine serum albumin (BSA) pH 7.4 (Sigma, St. Louis, MO, USA). After washing, cells were incubated with undiluted rat anti-mouse (anti-CD4, anti-CD8, anti-CD49b or anti-MAC-3) or hamster anti-mouse (anti-CD69) monoclonal antibodies (mAbs) specific for different cell surface markers and labeled with fluorescein isothiocyanate (FITC), phycoerythrin (PE) or peridinin chlorophyll-alpha protein (PerCP), all purchased from BD Biosciences Pharmingen (San Diego, CA, USA). Cell suspensions were homogenized and incubated for 30 min at room temperature in the dark. Cell surface-labeled samples were treated with FACS Lysing/fix Solution (BD Pharmingen), immediately vortexed and incubated at room temperature for 3 min in the dark. After the lysis/fixation procedure, membrane-labeled spleen cells (except for the samples incubated with anti-CD69-PE) were permeabilized for 10 min with FACS permbuffer (FACS buffer with 0.5% saponin, Sigma, St. Louis, MO, USA), washed and resuspended in FACS buffer containing the following antibodies: anti-IL-10, anti-IL-12p70, anti-IFN-γ or anti-TNF-α (BD Biosciences Pharmingen, San Diego, CA, USA). After intracytoplasmic staining, cells were washed with FACS buffer and were fixed with FACS FIX Solution (10 g/L paraformaldehyde, 1% sodium cacodylate, 6.65 g/l sodium chloride, 0.01% sodium azide). Data acquisition was performed in a Becton-Dickinson FACScalibur flow cytometer (BD Pharmingen, San Diego, CA, USA) with CELLQuest software provided by the manufacturer. A total of 30,000 (for only surface labeling) or 50,000 (for intracellular cytokines) events per tube were acquired. Flow cytometry analyses were performed using CELLQuest software, and the absolute number of each spleen cell subtype per spleen was determined.

### Statistical analyses

All statistical analyses were performed using GraphPad Prism 5.00 (GraphPad Software, San Diego, California, USA). The parameters studied, except survival analysis, were analyzed by One Way Analysis of Variance, and when differences between groups were verified, multiple comparisons were performed by the Student-Newman-Keuls' post-test. Survival analysis was carried out using the Kaplan-Meier method, and the significance of differences between groups was assessed using the logrank test. *P*-values of 0.05 or less were considered significant. The results were expressed as mean ± SEM.

## Results

### Course of infection

**Figure 2:**
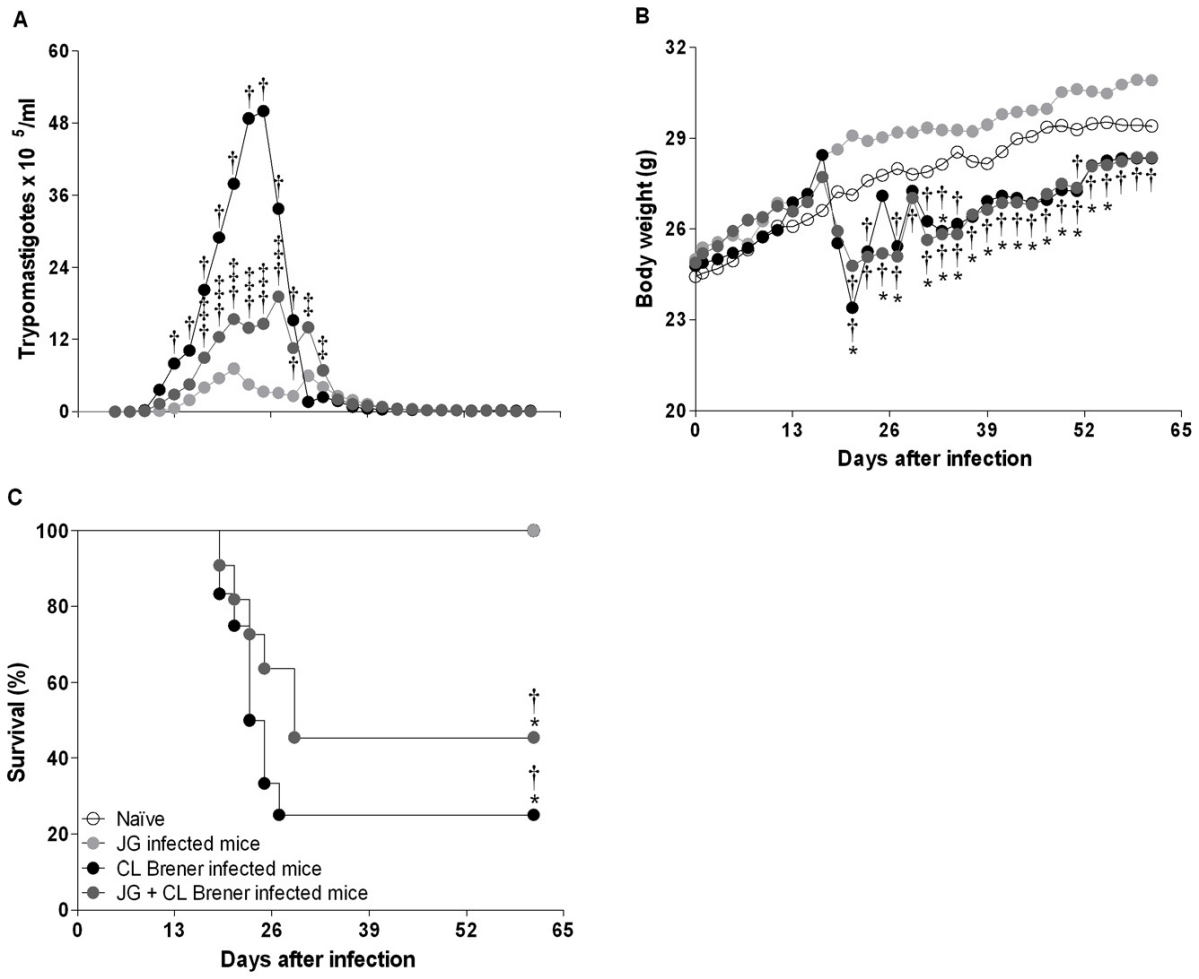
Assessment of the parasitemia, body weight and survival evolution. BALB/c mice were intraperitoneally infected with 100 trypomastigote forms of JG or CL Brener *T. cruzi* strains (single infection) or coinfected with 100 trypomastigotes of both *T. cruzi* populations in a 1∶1 ratio and the parasitemia (A), body weight (B), and survival (C) were assessed at different days post infection. Parasitemia levels and body weight are expressed as the arithmetic mean of six or twelve mice per group (representative of two independent experiments). Symbols as follows: white circle: Naïve mice; light gray circle: JG single infected mice; black circle: CL Brener single infected mice; and dark gray circle: JG and CL Brener coinfected mice. *, † and ‡ represent *P*<0.05 when compared with naïve animals, JG single infected and CL Brener single infected mouse groups, respectively.

BALB/c mice were infected with 100 trypomastigotes of JG or CL Brener (single infection) or coinfected with 100 trypomastigotes derived from a recently prepared mixture of both *T. cruzi* populations in a 1∶1 proportion via intraperitoneal route. The parasitemia levels, assessed from the 5^th^ day p.i. to the time point that the parasites became undetectable, revealed that JG single infected mice presented lower parasitemia in relation to all other infected animal groups in spite of the day p.i. evaluated. In contrast, animals single-infected with CL Brener presented higher parasitemia, while mice coinfected with JG and CL Brener presented intermediate levels of parasitemia (Figure 2A). This behavior cannot be explained by the simple effect of the relative reduction in the CL Brener inoculum from 100 trypomastigotes (used in the single infections) to 50 trypomastigotes (used in the double infection), since mice single-infected with 50 trypomastigotes of CL Brener present similar parasitemia, symptoms and survival curve to those observed among mice single-infected with 100 trypomastigotes forms of CL Brener (data not shown).

Body weight loss was more significant among mice infected with CL Brener alone or in the presence or absence of JG in relation to other groups. This was especially noteworthy on the 21^st^ day p.i., when body weights among CL Brener single infected (22.8±1.4 g) or coinfected (24.0±0.9 g) mice were significantly lower in relation to naïve (27.0±0.4 g) or JG single infected (29.0±0.5 g) animals. However, the body weights of all infected mice that overcame the acute phase of disease returned close to those of naïve mice during the course of infection (Figure 2B). Similar to the parasitemia and body weight loss levels, the mortality rate was null among naïve or single JG infected mice, higher among CL Brener single infected mice (75%) and intermediary among the coinfected mice (55%). However, despite slight differences observed in the mean survival time among CL Brener single infected (24±3 days p.i.) and coinfected (29±3 days p.i.) mice, the survival curves of animals infected with CL Brener alone or in the presence of JG were not significantly different (Figure 2C).

### Changes in hematological parameters during the acute phase of infection

**Figure 3:**
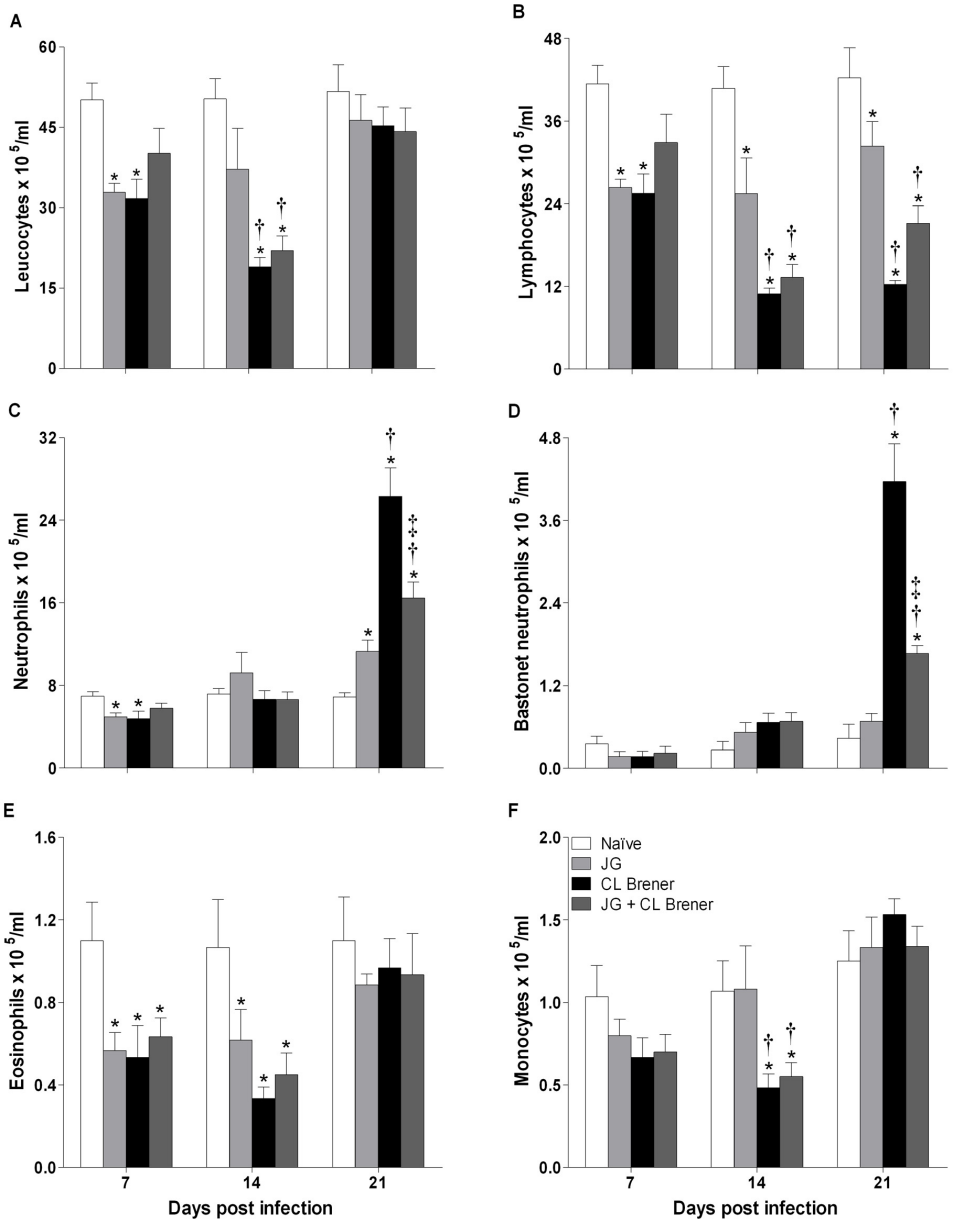
Total and differential blood leukocyte counts from BALB/c mice infected with JG and/or CL Brener. Groups of mice were infected with 100 trypomastigotes of JG or CL Brener (single infection) or coinfected with 100 trypomastigotes of both *T. cruzi* populations in a 1∶1 ratio via the intraperitoneal route, and blood leukocyte counts were assessed at 7, 14 and 21 days p.i. Symbols as follows: (A) leucocytes; (B) lymphocytes; (C) neutrophils; (D) bastonet neutrophils; (E) eosinophils; (F) monocytes; white bar: Naïve mice; light gray bar: JG infected mice; black bar: CL Brener infected mice; and dark gray bar: JG and CL Brener infected mice. Values are expressed as the mean ± SEM of three mice per group (representative of two independent experiments). *, † and ‡ represent *P*<0.05 when compared with naïve, JG and CL Brener mouse groups, respectively.

Peripheral blood samples were analyzed to assess the effects of JG and/or CL Brener infection on hematological parameters. There was a significant reduction in global leukocyte numbers on days 7 and 14 p.i. among CL Brener infected mice, in the presence or absence of JG. Infection with JG alone led to reduction only on day 7 p.i. (Figure 3A). Differential leukocyte counts also revealed variation among the groups of infected mice (Figure 3B–F). A significant reduction in lymphocyte counts was observed for all groups, but the magnitude of lymphopenia was more intense among CL Brener single infected mice (Figure 3B). Neutrophilia (Figure 3C) and bastonet neutrophilia (Figure 3D) were observed at day 21 p.i. among CL Brener infected animals. JG infected mice presented almost normal neutrophil and bastonet neutrophil counts, while coinfected animals presented intermediary counts (Figure 3C and 3D). Significant eosinopenia was observed in all infected mice on 7^th^ and 14^th^ days p.i., but returned to basal levels by 21 days p.i. (Figure 3E). Regarding monocyte counts, significant reduction was only observed on the 14^th^ day p.i among animals infected with CL Brener in the presence or absence of JG (Figure 3F). Besides leukocyte amounts, the hemoglobin level, hematocrit and red blood cell concentration were also determined. A significant reduction in these parameters was only observed on the 21^st^ day p.i. and only among animals infected with CL Brener in the presence or absence of JG (Table 2). Platelet counts were not significantly different among experimental groups during the course of infection (data not shown).

**Table 2:** Hemoglobin level, hematocrit and red blood cell count in JG and/or CL Brener infected mice at 21 days p.i.

*Experimental groups* *	*Haemoglobin (g/dl)*	*Haematocrit (%)*	*Red blood cell count (×10^9^/ml)*
Naïve	15.08±0.64	42.97±2.14	8.95±0.33
JG	14.42±0.61	41.27±2.20	8.78±0.41
CL Brener	10.85±0.31*†	32.07±1.07*†	7.00±0.12*†
JG + CL Brener	11.88±0.26*†	34.85±0.99*†	7.48±0.20*†

*: Groups of mice were infected with 100 trypomastigotes of JG or CL Brener (single infection); or coinfected with 100 trypomastigotes of both *T. cruzi* populations in a 1∶1 proportion via intraperitoneal route, and haemoglobin level, hematocrit determination and red blood cell count were assessed at 7, 14 and 21 days p.i. Values are expressed as the mean ± SEM of three mice per group (representative of two independent experiments).

*: and ^†^represent *P*<0.05 compared with naïve and JG mice groups, respectively.

### Heart histopathology, tissue parasitism and morphometric analysis

**Figure 4:**
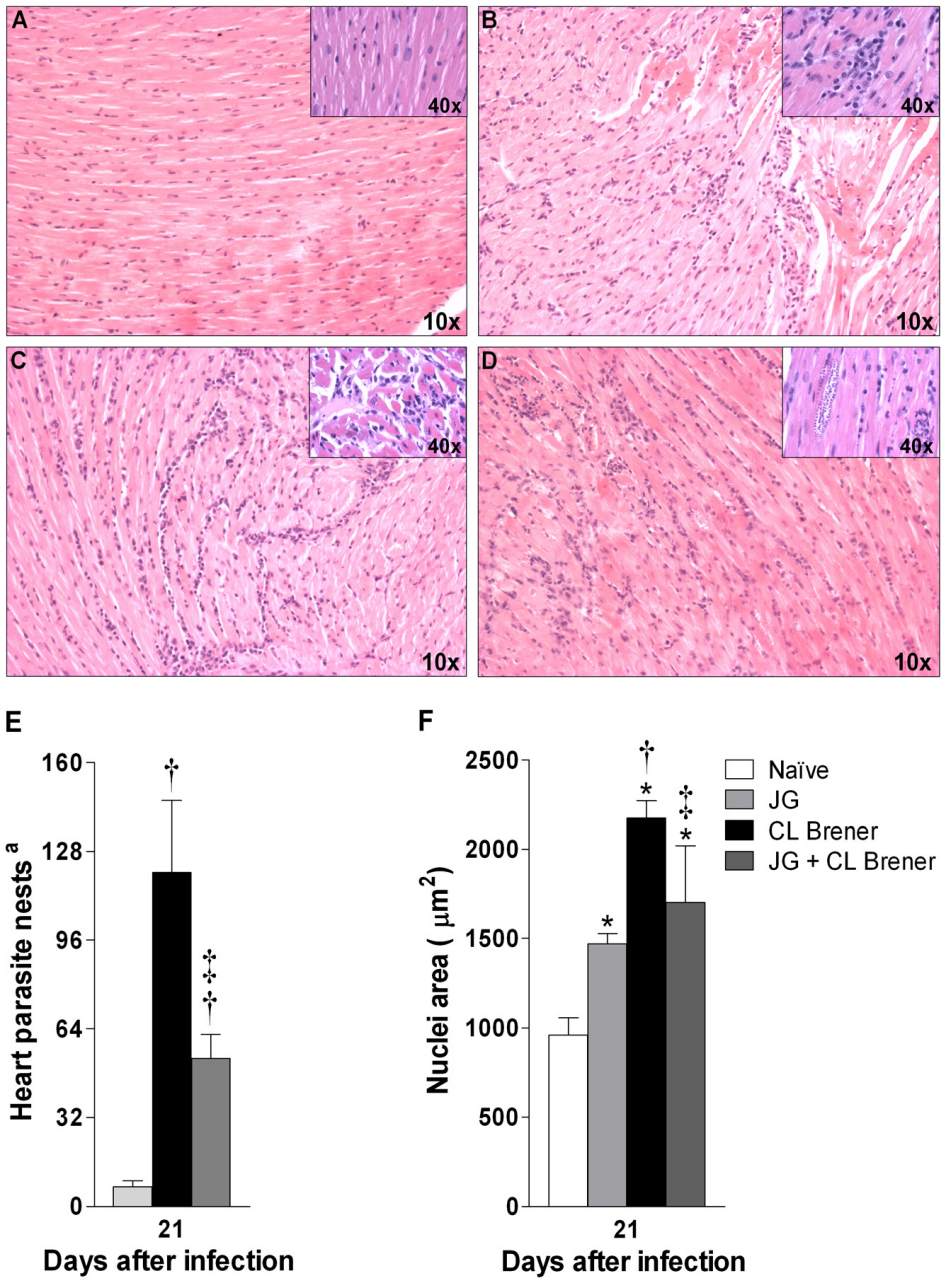
Heart histopathology, morphometric analysis and parasitism in BALB/c mice infected with JG and/or CL Brener. HE-stained representative myocardial sections from mice infected with 100 trypomastigotes of JG or CL Brener (single infection) or coinfected with 100 trypomastigotes of both *T. cruzi* populations in a 1∶1 ratio via the intraperitoneal route were collected at 21 days p.i. (A–D) and were histopathologically analyzed in original magnifications of 10× or 40× (details). Total number of parasite nests (E) and nucleus area quantification (cellularity) were assessed using computer-aided morphometry (F), Symbols as follows: A or white bar: Naïve mice; B or light gray bar: JG infected mice; C or black bar: CL Brener infected mice; and D or dark gray bar: JG and CL Brener infected mice. ^a^Represents number of parasite nests counted in three semi-consecutive sections. Values are expressed as the mean ± SEM of three mice per group (representative of two independent experiments). *, † and ‡ represent *P*<0.05 when compared with naïve, JG and CL Brener mice groups, respectively.

Parasite-induced cell destruction followed by focal inflammation usually correlates to tissue damage and heart malfunction. We evaluated heart inflammatory infiltrates and parasitism to assess the differential effects of infection with JG and/or CL Brener in heart tissue lesions at 7, 14 and 21 days p.i. As expected, JG and/or CL Brener infected mice presented typical heart histopathological alterations of the acute phase of infection, such as inflammatory infiltrates predominantly constituted by mononuclear cells, edema and some degree of degenerative changes of the myocardium (Figure 4). At 7 days p.i., the heart inflammatory response was more intense among JG infected mice in relation to animals infected with CL Brener or coinfected, and the inflammatory foci, when present, were small (data not shown). At the 14^th^ day p.i., in all heart samples we noticed moderate inflammatory foci but we did not observe significant differences among infected mice (data not shown). Parasite nests were not visible yet. At the 21^st^ day p.i., however, hearts from CL Brener infected mice presented more intense and diffuse inflammation in the ventricular and atrial walls when compared to JG infected mice, as well as when compared to coinfected animals (Figure 4A–D and 4F). The acute myocarditis induced by JG was predominantly focal and more restricted to the epicardial face of the myocardium (Figure 4B). Coinfected animals presented acute myocarditis of intermediary intensity (Figure 4D) when compared to JG or CL Brener single infected mice (Figure 4B, 4C and 4F). Furthermore, heart tissue parasitism, evaluated by counting the number of parasite nests in three HE-stained semi-conservative sections, was significantly higher among CL Brener single infected mice in comparison to animals infected with JG only or coinfected. This last group presented an intermediary level of heart parasitism at 21 days p.i. (Figure 4E). It is important to notice that the pattern of myocarditis induced by single infection with JG was rarely associated with tissue damage (see detail in Figure 4B). Meanwhile, the pattern induced by CL Brener was more often associated with cardiomyocyte degeneration associated to disrupted nests of parasites (see detail in Figure 4C) than that observed in hearts from coinfected mice, (see detail in Figure 4D).

### Differential tissue tropism and parasitism of the heart as assayed by PCR and microsatellite analyses

As expected in the acute phase of experimental *T. cruzi* infection, parasite kDNA was detected by PCR in heart tissue samples from all infected mice on the 14^th^ and 21^st^ days p.i. However, only scarce amounts of parasite kDNA were detected in a small number of animals on the 7^th^ day p.i. (data not shown). The differential tissue tropism of JG and CL Brener in heart tissue samples from coinfected animals was evaluated by analyzing both LSSP-PCR and polymorphic microsatellite locus profiles.

LSSP-PCR profiles of heart tissue samples obtained from coinfected mice revealed the presence of JG-specific amplicons (229, 233 and 258 bp) in 66% and 100% of samples collected on the 14^th^ and 21^st^ days p.i., respectively. The CL Brener-specific amplicon of 248 bp, in turn, was detected in 100% of samples collected on both the 14^th^ and 21^st^ days p.i. The relative amount of CL Brener/JG kDNA detected in the hearts analyzed varied from 76±9 to 77±8% in samples collected on the 14^th^ and 21^st^ days p.i., respectively.

Similar results were observed using *TcAAAT6* microsatellite locus analysis. Heart tissue samples obtained from coinfected mice revealed the presence of JG-specific alleles (271 and 275 bp) in 16% and 50% of samples collected on the 14^th^ and 21^st^ days p.i., respectively. The CL Brener-specific allele (263 bp) was detected in 100% of samples collected on both the 14^th^ and 21^st^ days p.i. The relative amount of CL Brener/JG kDNA detected in the heart samples analyzed varied from 97±3 to 93±3% in samples collected on the 14^th^ and 21^st^ days p.i., respectively.

### Serum cytokine and NO-derived metabolite levels during acute phase of infection

**Figure 5:**
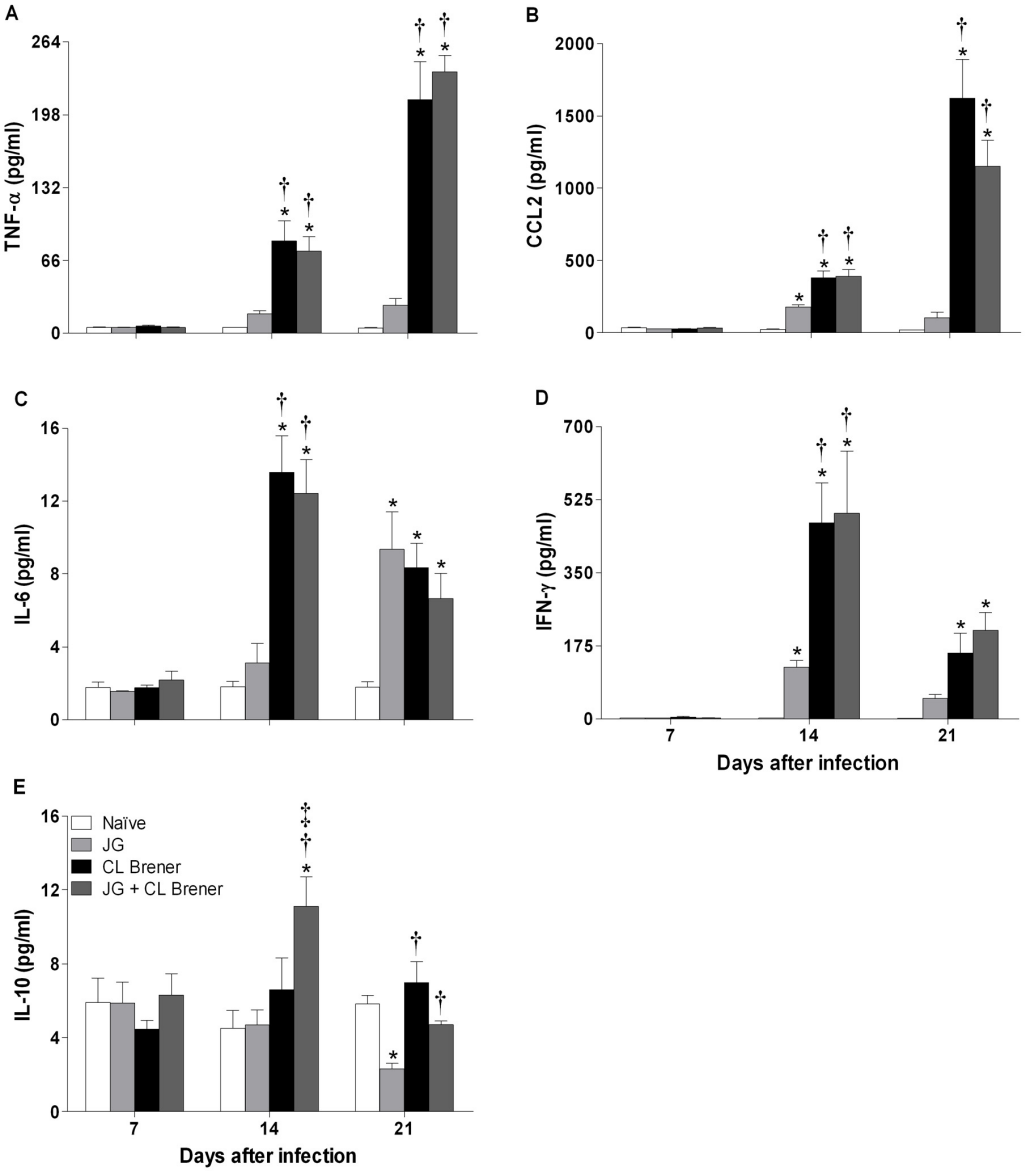
Serum cytokine levels in BALB/c mice infected with JG and/or CL Brener. Groups of mice were infected with 100 trypomastigotes of JG or CL Brener (single infection) or coinfected with 100 trypomastigotes of both *T. cruzi* populations in a 1∶1 ratio via the intraperitoneal route, and serum cytokine levels were assessed at 7, 14 and 21 days p.i. Symbols as follows: (A) TNF-α; (B) CCL2; (C) IL-6; (D) IFN-γ; (E) IL-10; white bar: Naïve mice; light gray bar: JG infected mice; black bar: CL Brener infected mice; and dark gray bar: JG and CL Brener infected mice. Values are expressed as the mean ± SEM of three mice per group (representative of two independent experiments). *, † and ‡ represent *P*<0.05 compared with naïve, JG and CL Brener mouse groups, respectively.

**Figure 6:**
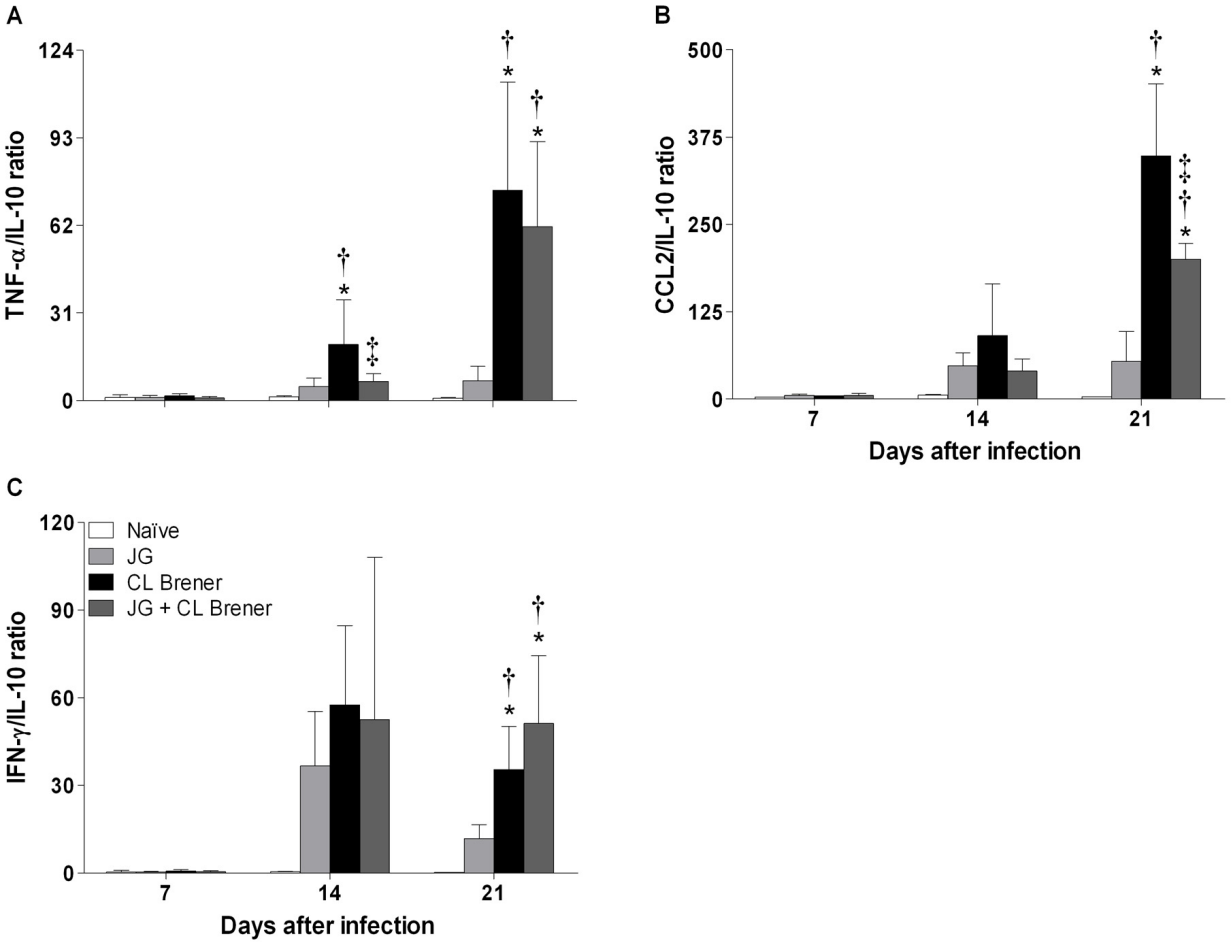
Serum TNF-α, CCL2 or IFN-γ to serum IL-10 ratios in BALB/c mice infected with JG and/or CL Brener. Groups of mice were infected with 100 trypomastigotes of JG or CL Brener (single infection) or coinfected with 100 trypomastigotes of both *T. cruzi* populations in a 1∶1 ratio via the intraperitoneal route, and TNF-α/, CCL2 or IFN-γ to serum IL-10 ratios were assessed at 7, 14 and 21 days p.i. Symbols as follows: (A) TNF-α/IL-10; (B) CCL2/IL-10; (C) IFN-γ/IL-10; white bar: Naïve mice; light gray bar: JG infected mice; black bar: CL Brener infected mice; and dark gray bar: JG and CL Brener infected mice. Values are expressed as the mean ± SEM of three mice per group (representative of two independent experiments). *, † and ‡ represent *P*<0.05 when compared with naïve, JG and CL Brener mouse groups, respectively.

To assess whether differences in the outcome of infection with the JG and/or CL Brener were associated with particular patterns of cytokine response, we determined the levels of cytokines (IL-2, IL-4, IL-5, IL-6, IL-10, IL-12p70, IFN-γ, CCL2 and TNF-α) and nitrite, a more stable NO-derived metabolite, in serum samples collected at 7, 14 and 21 days p.i. A slight increase in serum levels of IL-2 (naïve mice: 1.70±0.13 and CL Brener infected: 2.18^*^±0.11, mean ± SEM, ^*^ *P*<0.05) and IL-5 (naïve mice: 2.70±0.45 and CL Brener infected mice: 5.08^*^±0.38, mean ± SEM, ^*^ *P*<0.05) was detected only among CL Brener infected mice in relation to naïve mice and only on the 14^th^ day p.i. The biological significance of these small variations is unclear. Although measurable amounts of IL-4 and IL-12p70 were detected in serum samples from all experimental groups at all timepoints analyzed, no difference among groups was found (data not shown). Different patterns were observed, however, for all other measured cytokines. There were significant differences in serum levels of pro-inflammatory cytokines such as TNF-α, CCL2, IL-6 and IFN-γ at 14 and 21 days p.i. among CL Brener infected or coinfected mice in relation to naïve animals or JG single infected mice. It is noteworthy that IL-10 levels were maintained close to the basal level among infected animals on the 7^th^ and 21^st^ days p.i., while pro-inflammatory cytokine levels (TNF-α, CCL2, IL-6 and IFN-γ) presented a great variation among different experimental groups during the acute phase of infection (Figure 5A–E). Since cytokines act in a network of mutual interactions *in vivo*, ratios of pro-inflammatory cytokines (TNF-α, CCL2 and IFN-γ) to the immunoregulatory cytokine IL-10 were analyzed. The ratios of serum TNF-α, CCL2 or IFN-γ to serum IL-10 on the 7^th^, 14^th^ and 21^st^ days p.i. showed that animals infected with CL Brener or coinfected presented an increase in all TNF-α/, CCL2/ or IFN-γ/IL-10 ratios in at least one of three points analyzed in relation to both naïve mice and JG infected animals. However, coinfected animals had significant reductions in the ratio of TNF-α/IL-10 at 14 days, and of CCL2/IL-10 at 21 days p.i., suggesting a modulating role in the coinfection with JG and CL Brener in BALB/c mice (Figure 6A–C).

Regarding serum NO-derived metabolite levels, only NO_2_^−^ was measured in the present work and no significant difference between experimental groups at 7, 14 and 21 days p.i. was found (data not shown).

### Analysis of TNF-α- and IL-10-producing spleen cells

**Figure 7:**
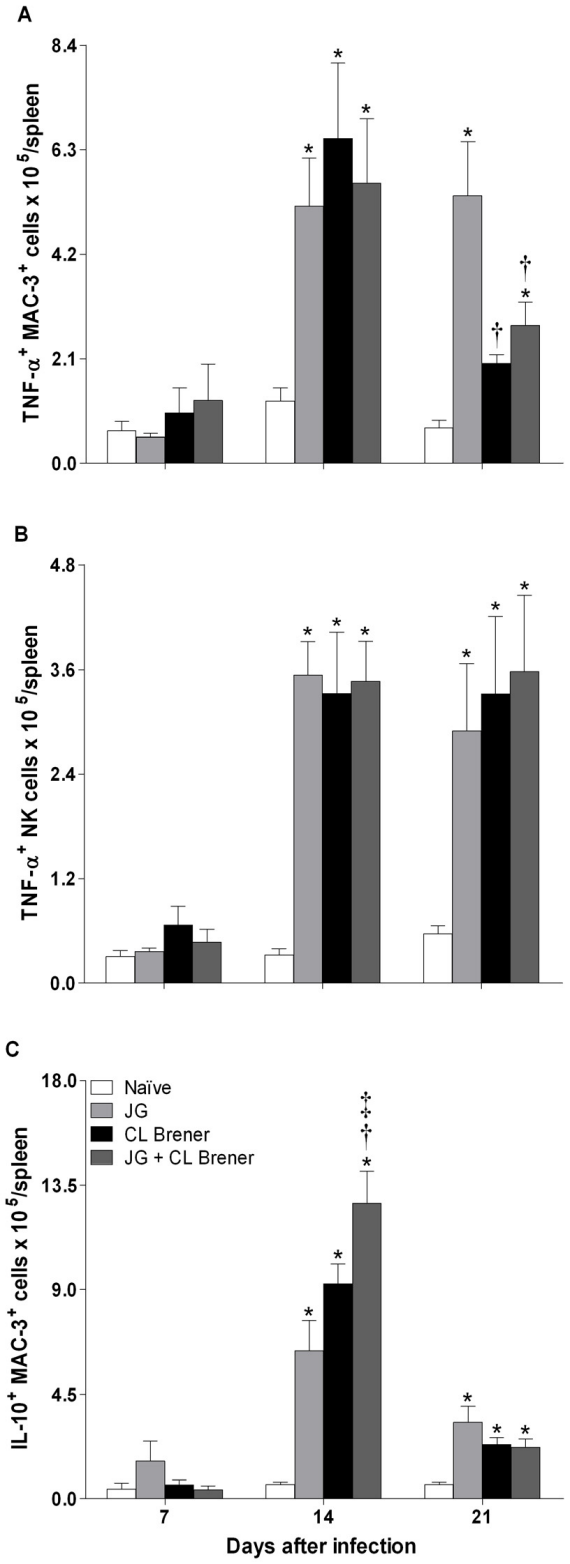
Flow cytometry analysis of splenic MAC-3^+^ and NK cells from BALB/c mice infected with JG and/or CL Brener. Groups of mice were infected with 100 trypomastigotes of JG or CL Brener (single infection) or coinfected with 100 trypomastigotes of both *T. cruzi* populations in a 1∶1 ratio via the intraperitoneal route, and flow cytometry analysis of splenic MAC-3^+^ and NK cells was performed at 7, 14 and 21 days p.i. Symbols as follows: (A) TNF-α^+^/MAC-3^+^; (B) TNF-α^+^/NK; (C) IL-10^+^/MAC-3^+^; white bar: Naïve mice; light gray bar: JG infected mice; black bar: CL Brener infected mice; and dark gray bar: JG and CL Brener infected mice. Values are expressed as the mean ± SEM of three mice per group (representative of two independent experiments). *, † and ‡ represent *P*<0.05 when compared with naïve, JG and CL Brener mouse groups, respectively.

**Figure 8:**
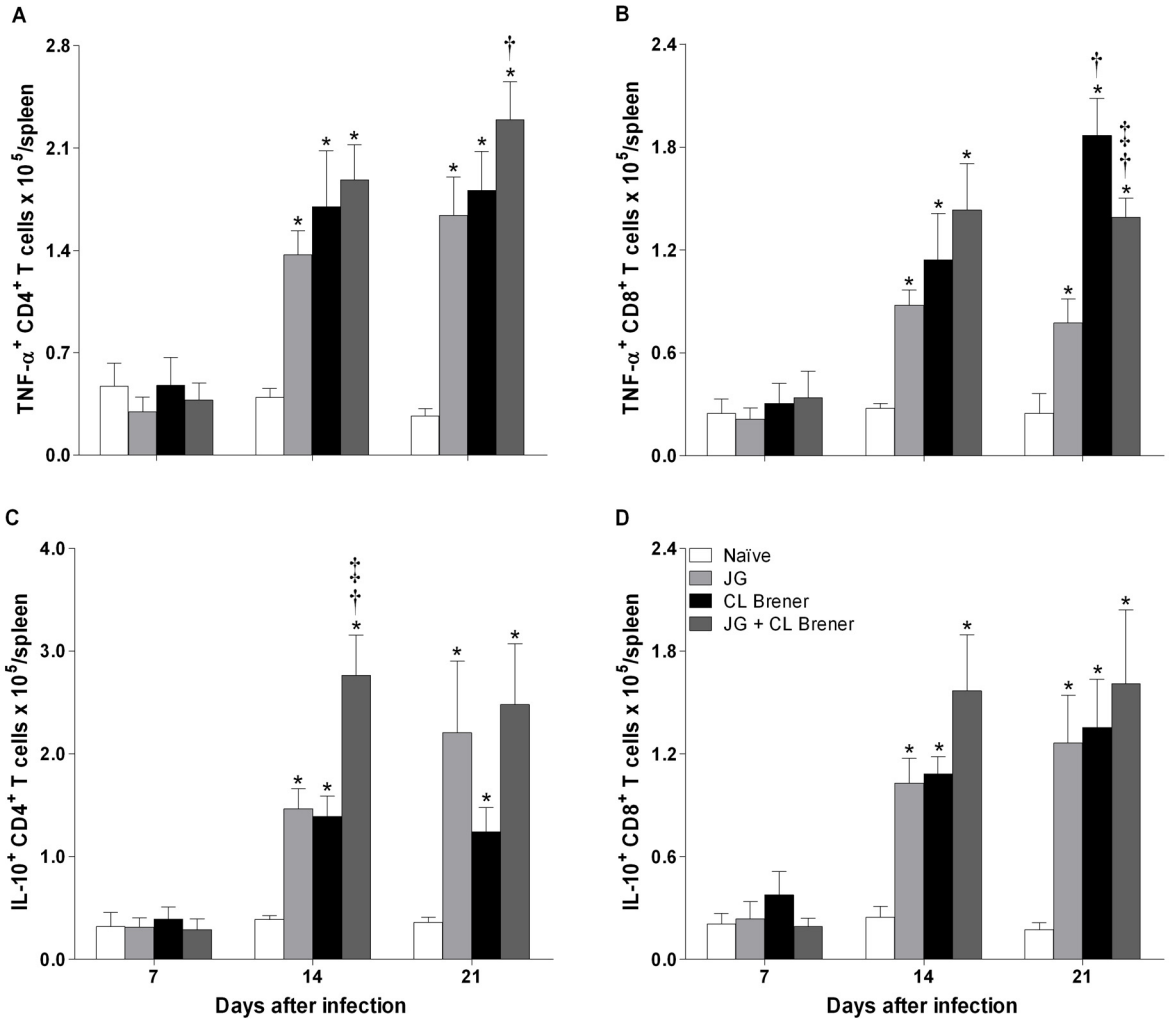
Flow cytometry analysis of splenic CD4^+^ T and CD8^+^ T cells from BALB/c mice infected with JG and/or CL Brener. Groups of mice were infected with 100 trypomastigotes of JG or CL Brener (single infection) or coinfected with 100 trypomastigotes of both *T. cruzi* populations in a 1∶1 ratio via the intraperitoneal route, and flow cytometry analysis of splenic CD4^+^ T and CD8^+^ T cells were performed at 7, 14 and 21 days p.i. Symbols as follow: (A) TNF-α^+^/CD4^+^; (B) TNF-α^+^/CD8^+^; (C) IL-10^+^/CD4^+^; (D) IL-10^+^/CD8^+^; white bar: Naïve mice; light gray bar: JG infected mice; black bar: CL Brener infected mice; and dark gray bar: JG and CL Brener infected mice. Values are expressed as the mean ± SEM of three mice per group (representative of two independent experiments). *, † and ‡ represent *P*<0.05 when compared with naïve, JG and CL Brener mice groups, respectively.

**Figure 9:**
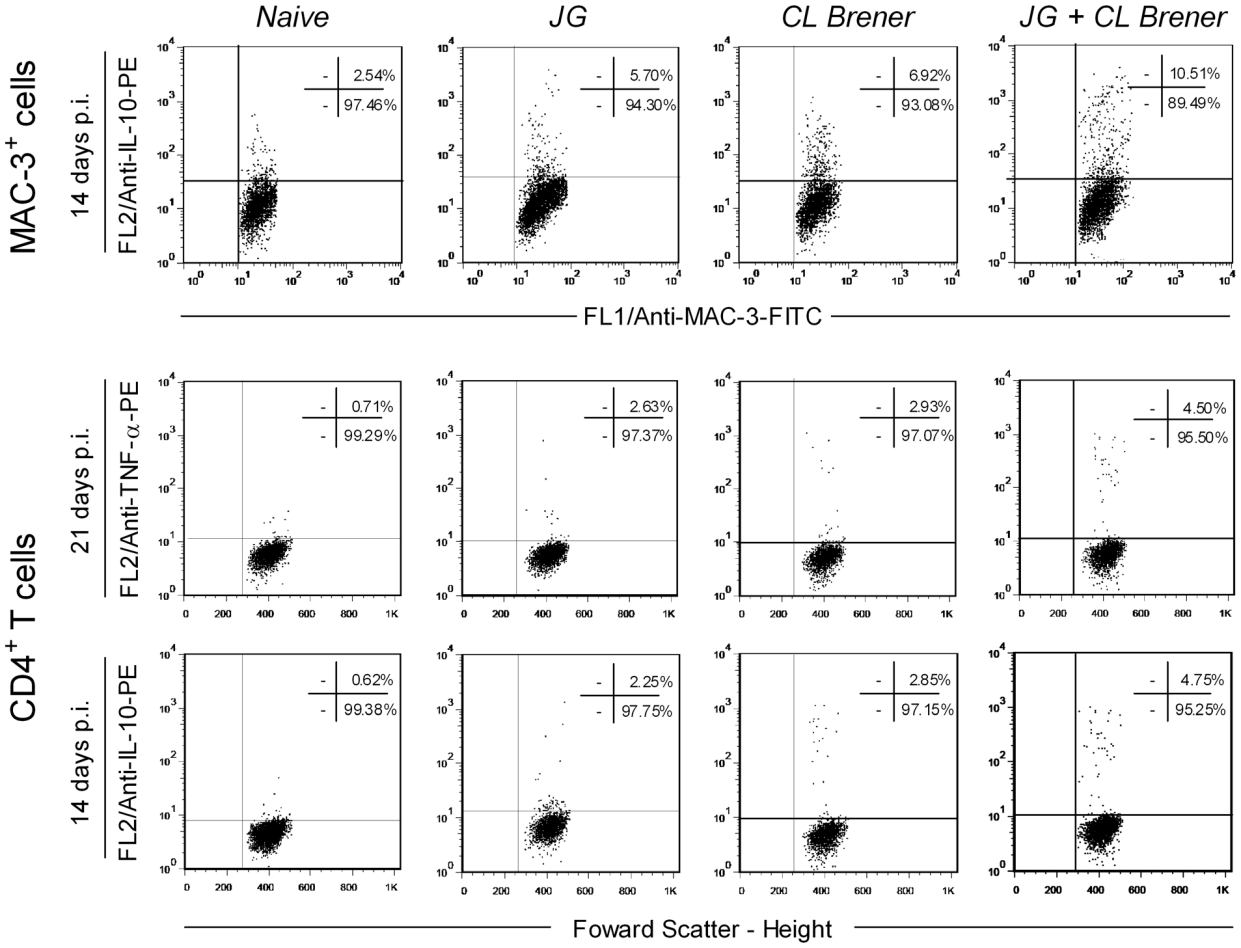
Representative flow cytometry charts illustrating the cytokine synthesis by spleen MAC-3^+^ and CD4^+^ T cells from BALB/c mice infected with JG and/or CL Brener. Results are presented in density plot format. The analyses were performed by quadrant statistics expressed as the percentage of cytokine^+^ cells within gated MAC-3^+^ at 14 days p.i., and CD4^+^ cells at 14 and 21 days p.i. in splenocytes cultures from naïve, JG single infected, CL Brener single infected and JG and CL Brener coinfected mice. Cytokine flow cytometry charts demonstrate the enhanced percentage of cytokine^+^ cells in all infected mice. Outstanding levels of TNF-α-producing CD4^+^ T cells were contra balanced by high frequency of IL-10-producing MAC-3^+^ and CD4^+^ T cells in coinfected mice.

Since the cytokines measured can be produced by more than one cell type, we evaluated next which cell type could be the source of the pro-inflammatory cytokine TNF-α and the immunoregulatory cytokine IL-10 in the spleen. These two cytokines were chosen because they represent opposite points on the inflammatory spectrum of immune response; the ratio between them revealed a significant difference between single infection with CL Brener and coinfection with JG and CL Brener. Analysis of TNF-α-producing cells showed that MAC-3^+^, NK, CD4^+^ and CD8^+^ T cells were important sources of this cytokine at days 14 and 21 p.i. for all infected mice (Figure 7A and 7B; 8A and 8B; 9). At day 21 p.i., the number of TNF-α-producing MAC-3^+^ cells was reduced in mice infected with CL Brener or coinfected (Figure 7A). However, the number of TNF-α-producing CD4^+^ and CD8^+^ T cells was augmented in coinfected and CL Brener infected mice, respectively (Figure 8A and 8B). Interestingly, the number of IL-10-producing macrophages and CD4^+^ T cells showed a significant increase in coinfected mice at day 14 p.i. (Figure 7C and 8C). In addition, no decrease in the number of these cell subpopulations was observed at any time point analyzed in coinfected mice.

## Discussion

The acute phase of CD is characterized by both high parasitemia and tissue parasitism, but most of the patients present few or no clinical symptoms in this phase of disease. Therefore, studies related to the early activation phase induced by natural *T. cruzi* infection in humans are scarce, and most information concerning parasite-associated features and host immunity related to *T. cruzi* infection is derived from studies using experimental models, in particular the murine model. Currently, we have a large amount of scientific information concerning the immune response during the early activation phase in animals acutely infected with *T. cruzi*, especially in BALB/c and C57BL/6 mice, which present different susceptibility to various intracellular pathogens, among them *T. cruzi* [25], [26]. However, most of these studies are either restricted to single-infected mice or are focused on analyses of few parasitological or immunological parameters. Co-existence of natural mixed infections among humans certainly plays an important role in the context of CD, and the complex interrelationships between host- and parasite-related factors might ultimately influence the outcome of infection with *T. cruzi*. Herein, we investigated the effects of the association of JG and CL Brener during the acute phase of infection in BALB/c mice by simultaneously analyzing different parasitological, histopathological and immunological parameters.

To better simulate natural infection conditions, inocula of 100 trypomastigotes of each parasite population (JG or CL Brener) or of a mixture of them (JG and CL Brener) were used. This inoculum is much smaller than those commonly used in the literature, which frequently reach 10^4^ trypomastigotes or more per animal. Assessment of parasitemia and heart parasitism revealed great differences in parasite burden between JG and/or CL Brener infected mice. JG single infected mice presented lower parasitemia and heart parasitism compared to the CL Brener infection, which induced high parasitemia and heart parasitism, at least in the acute phase of infection. Animals coinfected with JG and CL Brener presented levels of parasitemia and parasitism at an intermediate level compared to those from JG or CL Brener single infected mice. The significant reduction in heart parasite nests observed in coinfected animals when compared to CL Brener infected ones correlates with a decrease in the number of inflammatory cells (measured by nucleus area), suggesting that coinfected animals had a less intense inflammatory reaction in the heart. It is plausible that the reduction of these two important parameters contributes to the trend observed in the mortality curve showing improved survival of coinfected mice.

Direct identification of parasite populations in heart tissue samples from double-infected mice revealed a relative predominance of CL Brener, varying from 50 to 100% in all analyzed tissues depending on the technique and on the time elapsed since infection considered. Although the percentage of JG detected was always lower than CL Brener, we observed a progressive increase in the presence of JG in the heart samples from double-infected animals throughout the acute phase of infection. The relative predominance of one of the parasite population in the hearts of the animals seems to correlate to the genetic aspects of the parasites and the hosts, rather than to the initial inoculum used, since similar results were observed using a mixture containing 50 or 100 parasites of each parasite population. In addition, previous studies have demonstrated that variation of one component of the parasite mixture or of the mouse genetic background, especially MHC-associated genes, can interfere in the relative predominance of a parasite population in different tissues, as well as in disease evolution [3], [4], [9], [27], [28].

The severity of disease induced by *T. cruzi* infection in BALB/c mice was measured by the assessment of body weight loss, heart tissue damage and mortality rate, and it corresponded to the parasite population involved. Absence of evident symptoms of disease, moderate acute myocarditis and null mortality were observed among JG single infected mice. On the other hand, CL Brener single infected mice presented gradual and progressive disease, characterized by anorexia, lethargy and cachexia, as well as severe acute myocarditis and a high mortality rate. Interestingly, coinfected animals presented symptoms similar to those presented by CL Brener infected mice, yet with lower magnitude. In addition, these animals presented less heart tissue damage, a reduced mortality rate and longer mean survival time compared to CL Brener single infected mice. It is interesting that Franco *et al.* (2003), working with the same *T. cruzi* populations but a different host, observed similar results [8].

Regardless of the experimental model studied, the inflammatory response triggered by infection or tissue damage involves the coordinated recruitment of blood components (plasma and leukocytes) to the site of infection or injury. The relative and absolute numbers of peripheral blood cells are critically regulated in physiological conditions, and disruptions in this physiological balance can be clinically detected in several disease states. In accordance with this, we found considerable variations in blood leukocyte counts among *T. cruzi* infected animals. The leukopenia observed among infected mice during the early phase of infection is probably caused by an intense recruitment of leukocytes to the inflammatory sites, and the return of total leukocyte counts close to basal levels at 21 days p.i. was mainly related to a significant increase in neutrophil counts. More importantly, we observed a severe and persistent lymphopenia among CL Brener single infected or coinfected animals at 14 and 21 days p.i., a condition that can be associated with both an immunosuppressive state and the high mortality rates observed among these animals during the course of infection. Marcondes *et al.* (2000) reported severe hematological alterations, characterized by pancytopenia and a low number of bone marrow blood cell precursors, in particular erythroblasts and megakaryoblasts, in mice infected with *T. cruzi*. Infection was accompanied by anemia, decrease in hematocrit and hemoglobin levels, as well as an exponential growth of parasites, and high mortality [29]. In the present study, we showed a significant anemia, with decreases in both number of red blood cells and hematocrit, as well as in hemoglobin levels among animals single infected with the CL Brener or coinfected at 21 days p.i., which may contribute to high mortality rates among these mice. The lifespan of murine red blood cells is from 1 to 2 months; anemia was detected earlier than this. Therefore, reduced lifespan of red blood cells should be considered as an additional factor that contributes to anemia, which can influence survival of *T. cruzi* infected hosts.

*T. cruzi* is capable of infecting a wide variety of host cells, but the persistence of this parasite in cardiac, skeletal and smooth muscle cells is, at least in part, a key aspect of both the chronic phase of the infection, as well as the outcome of disease. The first step to ensure *T. cruzi* survival and successful infection is to enter host cells. Several molecules present on host cells and on the parasite surface are essential for the process of cell invasion [30] and are capable of stimulating an innate immune response upon the first encounter [31]–[33]. These early interactions are critical for immediate control of parasitemia and parasitism, as well as for establishment of a cytokine-rich microenvironment that influences the generation and direction of the downstream adaptive immune response. With this in mind, we scrutinize the host response to *T. cruzi* infection through analysis of immunological parameters, such as serum cytokine and NO levels, as well as analysis of the expression profile of cytokines by several spleen cell subpopulations.

High levels of serum TNF-α during the course of disease are usually associated with toxemia symptoms (anorexia, lethargy and cachexia) and high mortality rates. TNF-α, also known as cachectin, is produced primarily by mononuclear phagocytes (monocytes and macrophages) and acts as a multipotent modulator of immune responses; it is also a potent endogenous pyrogen, a well-known mediator of cachexia, and a marker of sepsis. Due to its multiple functions in immunological activity, TNF-α plays a critical role in several conditions that involve systemic inflammatory responses, such as sepsis and toxic shock [34], [35]. In accordance with this, animals single-infected with JG presented serum TNF-α levels similar to those found in naïve mice, and no clear symptoms of disease. Hölscher *et al.* (2000) showed that the TNF-α neutralization not only attenuated disease progression, but also prolonged the survival of IL-10^−/−^ mice infected with *T. cruzi*. Taking these findings together, it is reasonable to assume that TNF-α can be the direct mediator of mortality due to a toxic shock-like systemic inflammatory response observed among animals infected with CL Brener and, to a lesser extent, with both strains (coinfected).

At the same time, it is widely recognized that inflammatory responses have a critical role in protection against infection, though they may contribute to the pathology of it. Therefore, to avoid pathological side effects, the inflammatory reaction induced during immune responses must be tightly regulated. In tune with this, wild-type C57BL/6 mice infected with *T. cruzi* survived, but IL-10^−/−^ mice with the same genetic background presented a high mortality rate, despite presenting low parasitemia levels and high systemic production of pro-inflammatory cytokines (IFN-γ, IL-12, and TNF-α) during the acute phase of infection [34], [36]. These findings show that IL-10, an anti-inflammatory cytokine, has a critical role in control of the immune response during experimental *T. cruzi* infection. In this study, we observed that although coinfected animals presented high levels of serum TNF-α during the acute phase of infection, the potential toxic effects of TNF-α were counterbalanced by the production of significant serum levels of IL-10. This resulted in a low and significant TNF-α/IL-10 ratio that may have contributed to the lower mortality rate and to the higher survival time observed among coinfected animals In fact, there are several reports on the immuno-modulatory role of IL-10 in infectious diseases including Chagas disease. In canine infection by *T. cruzi*, the development of chronic cardiomyopathy correlates with high levels of IFN-γ and TNF-α and low levels of IL-10 [37]. In human Chagas disease as well the presence of a polymorphic allele of IL-10 gene, which results in lower expression of this cytokine is associated with cardiomiopathy and a severe form of the disease [5]. Moreover, a study on cerebral malaria showed recently that coinfection of mice with non-lethal *Plasmodium berghei* XAT suppressed experimental cerebral malaria caused by infection with *Plasmodium berghei* ANKA. The modulatory effect of the coinfection was abolished in IL-10-deficient mice clearly showing the central role of IL-10 in inhibiting the inflammatory cytokines IFN-γ and TNF-α involved in brain damage [38].

In addition to the reduction in the ratio of TNF-α to IL-10, we also found a significant decrease in the CCL2/IL-10 ratio in serum samples from animals infected with CL Brener in the presence of JG. CCL2 (MCP-1) is another important pro-inflammatory mediator characterized as a monocyte-specific chemoattractant that also attracts NK cells and T lymphocytes. It is mainly produced by macrophages in response to a wide range of cytokines such as IL-6, TNF-α and IL-1β, but can upon stimulation also be produced by a variety of cells, such as fibroblasts and endothelial cells. CCL2 is secreted in the course of *T. cruzi* infection and participates in *T. cruzi* uptake and activation of trypanocidal activity in macrophages. Paiva *et al.* (2009) showed that mononuclear cells from *T. cruzi*-infected CCL2^−/−^ mice (in contrast to WT mice) do not form heart focal infiltrates. In this case, the parasite burden is greater, and tissue infiltrates are composed of less-activated CD8 lymphocytes and macrophages, which are essential to control parasite growth [39]. In the present study, we found high levels of CCL2 among mice single-infected with CL Brener, which can explain, at least in part, the intense myocarditis characterized by inflammatory infiltrate (predominantly constituted of mononuclear cells) observed among these animals. Reduction in the CCL2/IL-10 ratio in the mice may have also contributed to controlling the inflammatory reaction in the heart and the improved survival of the coinfected mice.

Therefore, our results suggest that production of IL-10, a key element in the control of tissue damage triggered by exacerbated inflammatory response during the course of infection, elicited by coinfection with JG and CL Brener may have an important role in modulation of heart inflammation and survival. Flow cytometry analysis of spleen cell subpopulations producing IL-10 revealed that frequency of IL-10-producing MAC-3^+^ and CD4^+^ T cells were both elevated in coinfected mice when compared to single-infected ones.

In conclusion, our work reinforces that differential outcomes of *T. cruzi* infection can be influenced by the complexity of the infecting *T. cruzi* population and parasite load, as well as by factors related to regulation of acute inflammatory response that are essential for protection against infection, but may also contribute to pathology.
